# The Effect of Lycopene Preexposure on UV-B-Irradiated Human Keratinocytes

**DOI:** 10.1155/2016/8214631

**Published:** 2015-11-17

**Authors:** Andreia Ascenso, Tiago Pedrosa, Sónia Pinho, Francisco Pinho, José Miguel P. Ferreira de Oliveira, Helena Cabral Marques, Helena Oliveira, Sandra Simões, Conceição Santos

**Affiliations:** ^1^Instituto de Investigação do Medicamento (iMed.ULisboa), Faculdade de Farmácia, Universidade de Lisboa, Avenida Professor Gama Pinto, 1649-003 Lisboa, Portugal; ^2^Departamento de Biologia, Laboratório de Biotecnologia e Citómica, CESAM, Universidade de Aveiro, Campus Universitário de Santiago, 3810-193 Aveiro, Portugal

## Abstract

Lycopene has been reported as the antioxidant most quickly depleted in skin upon UV irradiation, and thus it might play a protective role. Our goal was to investigate the effects of preexposure to lycopene on UV-B-irradiated skin cells. Cells were exposed for 24 h to 10 M lycopene, and subsequently irradiated and left to recover for another 24 h period. Thereafter, several parameters were analyzed by FCM and RT-PCR: genotoxicity/clastogenicity by assessing the cell cycle distribution; apoptosis by performing the Annexin-V assay and analyzing gene expression of apoptosis biomarkers; and oxidative stress by ROS quantification. Lycopene did not significantly affect the profile of apoptotic, necrotic and viable cells in nonirradiated cells neither showed cytostatic effects. However, irradiated cells previously treated with lycopene showed an increase in both dead and viable subpopulations compared to nonexposed irradiated cells. In irradiated cells, lycopene preexposure resulted in overexpression of *BAX* gene compared to nonexposed irradiated cells. This was accompanied by a cell cycle delay at S-phase transition and consequent decrease of cells in G0/G1 phase. Thus, lycopene seems to play a corrective role in irradiated cells depending on the level of photodamage. Thus, our findings may have implications for the management of skin cancer.

## 1. Introduction

Human skin is constantly exposed to the UV irradiation that may induce a number of pathobiological cellular changes. Through lipid peroxidation, protein cross-linking, and DNA damage, UV-A and UV-B radiation (UVR) can cause photoaging and photocarcinogenesis [1–3]. Skin has a variety of enzymatic and small molecular antioxidants that can inhibit oxidative damage. However, the excessive ROS production often exceeds the skin antioxidant ability [4]. In this regard, emphasis on developing novel preventive and therapeutic strategies based on phytocompounds capable of ameliorating the adverse effects of ROS has become an important area of research. Moreover, primary prevention approaches of skin cancer proved to be inadequate in lowering the incidence of this type of cancer, emphasizing the need to develop novel skin cancer chemopreventive agents. Among the vast number of photochemoprotective agents, botanical antioxidants have given promising results [4]. Two types of chemopreventive agents could be useful for the management of skin cancer. Primarily, the agents that could inhibit the damage caused by UVR may prevent the formation of initiated cells (cells with cancerous potential). Secondly, the agents that could eliminate the initiated cells may reduce the risk of skin cancer [5].

Lycopene is a powerful antioxidant both* in vitro* and* in vivo* against the oxidation of proteins, lipids, and DNA, and it has been identified as one of the most potent scavengers of singlet species of oxygen free radicals—the highest among the carotenoids [6, 7]. At low oxygen tension, it can also scavenge peroxyl radicals, inhibiting the process of lipid peroxidation [8]. Lycopene was reported as the most quickly depleted antioxidant in skin upon exposure to solar radiation [9] and might play a role of protection against UVR. Recent research has been developed to assess if lycopene has potential for prevention of skin cancer. In fact, lycopene has been shown to inhibit proliferation of several types of cancer cells through different mechanisms in* in vitro* systems [10, 11]. Chemopreventive antioxidants are mostly studied for their role as radical scavengers, but this preventive role can be complemented by a corrective activity as selective inducers of apoptosis in transformed cells [12]. Moreover, Ribaya-Mercado et al. [9] suggested a role of lycopene in mitigating photooxidative damage in tissues.

Keratinocytes are the predominant cell type (95%) in the epidermis, the outermost layer of the skin [13]. Considering that the principal site of action of UV-B is the epidermis layer [14], keratinocytes might be more susceptible to UV-B-induced apoptosis than fibroblasts which are located in dermis layer (reached by UV-A) [15]. However, keratinocytes may be more UV-B resistant in terms of their proliferative ability as measured by colony survival assays and have greater ability for UV-DNA repair [15].

To date, most of the studies on the therapeutic potential of lycopene have been performed* in vivo* [16, 17]. These studies may be obscured by the complexity of biological system models.* In vitro* conditions may circumvent some of these contingencies and complement* in vivo* data within the 3Rs perspective (*Refine, Replace, *and* Reduce*). Despite the lower complexity of* in vitro* systems, the study of cellular photoprotection by antioxidants could be challenging because of the high chemical instability (especially to air and light) and strong lipophilicity of many antioxidant molecules such as lycopene. According to Zefferino et al. [11]* in vitro* experiments may occasionally produce inconsistent results due to lycopene's poor solubility in cell culture media [18]. In fact, lycopene is very hydrophobic (log *P* ≈ 15) and is usually solubilized in organic solvents such as tetrahydrofuran (THF). However, an uncontrolled precipitation process may occur upon addition to aqueous media, besides the high toxicity associated with these solvents. The solubility and uptake of these large crystals in the cells are quite limited and there is almost no protection against chemical degradation [19]. Alternative ways of delivering lipid-soluble compounds include micelles, microemulsions, nanoparticles, water-dispersible beadlets, artificial liposomes, enriched bovine serum, or other formulations, each of which has an influence on the cellular uptake and compounds stability [18, 20–23]. According to Palozza et al., niosomes provide a suitable, safe, and low-cost vehicle for *β*-carotene in cell culture [24]. Lipid-based delivery systems also show UV-blocking effects dependent on lipid composition and the particle size (the smaller the particle size, the higher the sunscreen activity). Lipid matrices can act as sunscreen carriers and increase the sun protection factor obtained after topical application of UV absorbers (BaSO_4_, SrCO_3_, and TiO_2_) incorporated within these carriers because they provide a fixation medium for these pigments [25, 26]. However, the UV-blocking effect of the vehicle is not desired in this case, besides the difficulties of using these hydrophobic systems for cell culture studies.

The main limitations of different vehicles used for lycopene cell delivery are summarized on Table 1. Each vehicle provides specific advantages but also offers some limitations such as cytotoxicity, poor solubility, and crystallization in the cell medium [27].

In addition, the half-life of free lycopene in solution at 37°C is less than few hours. Thus, until an efficient method of solubilizing lycopene in aqueous buffers and cell culture media is developed,* in vitro* studies on the effects of lycopene on living cells will continue to show considerable variation between laboratories and cell lines and should be interpreted with caution [39].

Pfitzner et al. [18] have demonstrated that methyl-*β*-CD (M-*β*-CD) was an improved vehicle for the investigation of carotenoids and other lipophilic compounds in* in vitro* test systems, compared to organic solvents. Carotenoids-M-*β*-CD complex was superior concerning biological availability, missing cytotoxicity and presenting excellent stability when compared to other application forms such as organic solvents, mixed micelles, liposomes, or beadlets. At least, the solubilization with M-*β*-CD was easily and reproducibly achievable under routine laboratory conditions.

According to these literature references [18, 27] and preformulations studies, we decided to use another similar CD derivative, dimethyl-*β*-CD (DM-*β*-CD), to solubilize and stabilize lycopene for cell exposure experiments. Depending on the formulation and exposure conditions, lycopene has been shown to prevent cellular damage or otherwise to sensitize damaged cells leading to increased cell death. We aimed to study the effects of lycopene as sensitizer and inducer of cell death in UV-B damaged keratinocytes. Our hypothesis was that cells preexposed to lycopene would be more sensitized to death, in case of subsequent irreversible damage by UV-B. For this, the nontumorigenic keratinocyte cell line HaCaT [40] was used. HaCaT cells were preexposed to lycopene for sensitization and subsequently exposed to damaging UV-B irradiation. The effect of lycopene preexposure was analyzed by assays focused on cytotoxicity, genotoxicity, oxidative stress, and apoptosis.

## 2. Materials and Methods

### 2.1. Preparation of Lycopene Complex Solution

In order to avoid the use of organic solvents, lycopene (Extrasynthese, Genay, France, with a purity ≥ 98%, UV assay) was solubilized by complexation with dimethyl-beta-cyclodextrin (CD, degree of substitution: 1.8) which was a generous gift from Wacker (Stuttgart, Germany). Aqueous solutions of lycopene complexed with CD (1 : 4 molar ratio) were prepared under aseptic conditions within concentrations of 0, 5, 10, 15, and 20 *μ*M from a concentrated lycopene solution previously stirred with CD during approximately 48 h and sonicated 30 min (before use), always protected from light and air. Lycopene solutions were always freshly prepared under light and air protection and stored at −20°C (except the pure lycopene standard, stored at −70°C). The osmolarity of the sample containing the highest lycopene concentration was determined using an automatic osmometer (Knauer, Berlin, Germany).

When nonspecified, all higher grade reagents were from Sigma-Aldrich (St. Louis, MO, USA). All solutions were prepared using ultrapure water obtained in a MILLI-Q System from Millipore (Billerica, MA, USA).

### 2.2. Human Immortalized Keratinocytes (HaCaT) Cell Line

The HaCaT cell line was obtained from Cell Lines Services (CLS) (Eppelheim, Germany). Handling and culture of these cells were adapted to meet CLS protocol procedures. Cells were aseptically grown in Dulbecco's modified Eagle's medium (DMEM, no HEPES, no Pyruvate), high glucose, supplemented with 10% fetal bovine serum (FBS), 2 mM L-glutamine, 1% penicillin-streptomycin (10,000 U/mL), and 1% fungizone (250 U/mL) (Gibco, Life Technologies, Grand Island, NY, USA) at 37°C in a humidified atmosphere with 5% CO_2_.

### 2.3. HaCaT Cell Growth and Confluence under Normal Culture Conditions

The standard cell growth conditions were established after the analysis of HaCaT growth curves. HaCaT cells were seeded in a 6-well cluster plate (300,000 cells/well). Cell confluence and morphology were daily observed under microscope. After 3 days, cells were harvested by trypsinization according to CLS procedure. Briefly, the culture was rinsed twice with phosphate-buffered saline solution (PBS) without Ca^2+^ or Mg^2+^ (Gibco, Life Technologies, Grand Island, NY, USA) and then with PBS containing 0.05% EDTA to remove desmosomes and incubated at 37°C about 5–10 min. After this, EDTA solution was replaced by a 1 : 1 mixture of EDTA/trypsin-solution (final concentrations of 0.025% and 0.05%, resp.). The trypsinization was achieved after incubating at 37°C for 15–20 min. At this time, a double volume of complete culture medium (with FBS) was added to stop the detaching process and cells were suspended with stronger shaking, followed by the use of a syringe (21 G) because of its mechanical resistance. All cell manipulation and growth and subsequent exposure conditions were performed under strict aseptic procedures.

Cell density was calculated by counting with a hemocytometer (Neubauer Improved) under a phase-contrast microscope Nikon Eclipse 80i (Coyoacán, Mexico).

### 2.4. Selection of Exposure Conditions

#### 2.4.1. Selection of UV-Irradiation Dose

Ten UV-B lamps (Sankyo Denki G8T5E, Kanagawa, Japan) with a peak emission at 312 nm were used as the UV-B source. The UV-B irradiation was measured with a VLX 312 radiometer equipped with a UV-B sensor (Vilber Lourmat, Marne-la-Vallée Cedex, France). Around 7,000 cells/well were cultured in a 96-well cluster plate with complete medium. Twenty-four hours after, the cells were exposed to five different UV-B irradiation doses [based on related literature [41–45] of ~75, 150, 200, 225, and 325 mJ/cm^2^ (moderate-high to very-high dose)]. In order to prevent UV quenching, prior to irradiation, the cell culture medium was replaced by the same volume of PBS after two washing steps with PBS. After UV-B irradiation, cells were fed with fresh growth medium and incubated for 24 h. Cell metabolic activity was assessed as described below by the MTT assay (often used as viability method) in order to choose just one UV-B dose. Nonirradiated samples were used as negative control.

#### 2.4.2. Selection of Complexed Lycopene Dose

**Figure 1:**
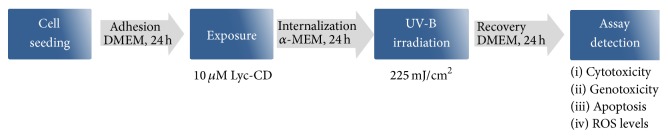
Culture conditions and experimental setting for the study of HaCaT cells exposed to complexed lycopene (Lyc-CD) and UV-B irradiation.

From previous cell viability results of HaCaT cells exposed to UV-B irradiation, new exposure conditions were tested with different Lyc-CD concentrations and a fixed UV-B irradiation dose. Briefly, cells were seeded and grown in DMEM (Dulbecco's modified Eagle's medium) supplemented with 10% FBS, 1% penicillin-streptomycin, and 1% fungizone, for 24 h, and then DMEM medium was replaced with *α*-MEM without nucleosides (Gibco, Life Technologies) with identical supplementation, containing complexed lycopene solutions (0, 5, 10, 15, and 20 *μ*M). Cells were exposed for 24 h, and after UV-B irradiation (225 mJ/cm^2^) HaCaT irradiated cells were incubated under standard culture conditions for a final 24 h period. The metabolic activity of cell culture was analyzed by MTT assay in order to choose just one lycopene dose for the subsequent analyses (Figure 1).

Briefly, for further experiments, HaCaT cells were seeded in a 6-well cluster plate (150,000–300,000 cells/2 mL well) and incubated 24 h under the same culture conditions, as mentioned above. After this period, the cells were exposed to complexed lycopene (10 *μ*M) for 24 h in order to achieve its cellular internalization and a higher cell confluence, and after UV-B irradiation (225 mJ/cm^2^) cells were allowed to grow under standard culture conditions for another 24 h to enable the occurrence of cellular repair mechanisms.

### 2.5. MTT Assay

Cell metabolic activity was assessed by the MTT (Sigma-Aldrich, St. Louis, MO, USA) assay, which is often used to roughly estimate culture's viability/proliferation characteristics. Cells were seeded at a density of 7,000 cells/well in a 96-well cluster plate. After complexed lycopene exposure, UV-B irradiation, and 24/h recovery the MTT assay was performed as previously described [46]. The optical density of reduced MTT was measured at 570 nm by spectroscopy on an automatic microtiter plate reader (Synergy HT Multi-Mode from BioTeK Instruments Inc., Winooski, VT, USA) and cell metabolic activity (MA ≈ viability or proliferation characteristics) was calculated according to

(1) Cell  MA%=Abs  570 nmsample−Abs  570 nmDMSOAbs  570 nmnegative  control−Abs  570 nmDMSO×100.

Besides the negative control (nonexposed and nonirradiated cells), the assays results were also compared to CD aqueous solutions (vehicle control) with the same dilution factors used for complexed lycopene samples.

### 2.6. Cell Cycle Analysis by Flow Cytometry

After complexed lycopene exposure and UV-B irradiation as described before, the cells were harvested with Accutase and centrifuged at 1157 g for 5 min at 4°C. The supernatant was removed and the cells were washed in PBS and suspended in 1 mL of 85% ethanol at 4°C and kept at −20°C until analysis. After that, the cells were centrifuged twice at 1157 g for 5 min at 4°C and suspended in 800 *μ*L PBS and vortexed. Cell cycle analysis was done as previously described [46]. Briefly, samples were filtered on a nylon mesh (50 *μ*m pore size) to the analysis tubes. Propidium iodide (PI), a DNA intercalating fluorochrome, and RNase were added to the samples (50 *μ*L each one) and vortexed. The mixture was incubated 20 min at room temperature and then analyzed by flow cytometry (FCM) using a Coulter EPICS XL flow cytometer. The instrument was equipped with an air-cooled argon-ion laser tuned at 15 mW and operating at 488 nm for excitation. Data was acquired using the SYSTEM II (v. 2.5) software. Integral fluorescence together with fluorescence pulse height and width emitted from nuclei was collected through a 645 dichroic long-pass filter and a 620 band-pass filter and converted on 1024 ADC channels. Prior to analysis the amplification was adjusted so that the peak corresponding to G0/G1 was positioned at channel 200. This setting was kept constant. The results were obtained in the form of three graphics: linear fluorescence light intensity (FL), forward angle light scatter (FS) versus side angle light scatter (SS), and FL pulse integral versus FL pulse height. This last cytogram was used to eliminate partial nuclei and other debris, nuclei with associated cytoplasm and doublets (these events have a higher pulse area but the same pulse height as single nuclei). Cell cycle analysis was performed using the FlowJo software (Tree Star Inc., Ashland, Oregon, USA) applying the Dean-Jett Model. Results were expressed as % of nuclei in G0/G1, S, and G2 phases of the cell cycle. The % of nuclei in Sub-G0/G1 indicative of DNA fragmentation was also computed.

### 2.7. Analysis of Apoptosis by Flow Cytometry

Early and late apoptosis were investigated by FCM using Annexin V-FITC Apoptosis Detection Kit from BD Pharmingen (Franklin Lakes, NJ, USA), as previously described [47]. Briefly, after complexed lycopene exposure and UV-B irradiation as described before, the cells were gently harvested with Accutase (PAA Laboratories, Pasching, Austria) and washed twice in cold PBS (1 mL). Finally, Annexin V-FITC and PI, 5 *μ*L of each, were added to 100 *μ*L of cell suspension (10^5^ cells/mL) in binding buffer. Samples were left in the dark for 15 min and 400 *μ*L of binding buffer was added. Samples were analyzed by FCM using a Coulter EPICS XL flow cytometer (Coulter Electronics, Hialeah, Florida, USA). Data were acquired using the SYSTEM II (v. 2.5) software. The cytogram of FITC fluorescence in log scale versus PI fluorescence in log scale allows the identification of nonapoptotic cells (Annexin V-FITC negative, PI negative), exclusively early apoptotic cells (Annexin V-FITC positive, PI negative), predominantly late apoptotic cells (Annexin V-FITC positive, PI positive), and predominantly necrotic or dead cells (Annexin V-FITC negative, PI positive). FCM data were analyzed by FlowJo software (Tree Star Inc., Ashland, OR).

### 2.8. RNA Extraction and qPCR Analysis of Apoptosis Regulating Genes

#### 2.8.1. Total RNA Extraction

At least 1 × 10^5^ HaCaT cells were rinsed twice with sterile PBS after removal of culture medium. After rinsing, cells were lysed in 1 mL TRIzol reagent (Life Technologies, Saint Louis, MO, USA) and after 5 min room temperature incubation, 200 *μ*L chloroform was added to each sample, shaken on vortex for 10 s, and incubated at room temperature for 2 min. Phase separation was achieved by centrifugation at 12,000 g for 5 min at 4°C in Phase-Lock Gel Heavy tubes (5 Prime 3 Prime, Inc., Boulder, CO, USA). The aqueous phase was mixed with 1 volume 70% ethanol and RNA was further purified using RNeasy Mini Kit columns (Qiagen, Hilden, Germany) following the manufacturer's recommendations. The total RNA was quantified by spectrophotometry at 260–280 and 230–260 nm (Nanodrop Spectrophotometer ND-1000, Thermo Fisher Scientific, Wilmington, DE, USA).

#### 2.8.2. cDNA Synthesis

1 *μ*g total RNA was reverse-transcribed using the Omniscript RT Kit (Qiagen, Hilden, Germany) in a reaction mixture containing 1 *μ*M Oligo(dT)18 primer, 5 mM dNTPs, reaction buffer, and RT enzyme according to the manufacturer's instructions. After the enzymatic reaction (incubation at 37°C for 1 h), cDNA samples were prediluted in milliQ water (1 : 20).

#### 2.8.3. Quantitative RT-PCR (qPCR)

For qPCR, primers were used complementary to the genes coding for Bax, Bcl-2, caspase 3, and TRAIL proteins, respectively, BAX, BCL2, CASP3, and TNFSF10. SDHA (succinate dehydrogenase) was used as the reference gene. The target genes and corresponding oligonucleotide primer sequences (5′ to 3′) were as follows:

BAX-F: GACGGCCTCCTCTCCTACTT; BAX-R: CAGCCCATCTTCTTCCAGAT; BCL2-F: GGAGGATTGTGGCCTTCTTT; BCL2-R: GCCGGTTCAGGTACTCAGTC; CASP3-F: GAACTGGACTGTGGCATTGA; CASP3-R: TGTCGGCATACTGTTTCAGC; TNFSF10-F (TRAIL-F): CCTGCAGTCTCTCTGTGTGG; TNFSF10-R (TRAIL-R): ACGGAGTTGCCACTTGACTT; SDHA-F: CTGCAGAACCTGATGCTGTGT; SDHA-R: GGATGGGCTTGGAGTAATCG.

Primer design was performed using Primer3 [48] and primer specificity was confirmed using the In-Silico PCR UCSC Genome Browser [49]. The final individual qPCR reactions contained iTaq Universal SYBR Green Supermix (BioRad, Hercules, CA), 150 nM of each primer, and 1 : 4 (v/v) prediluted cDNA (1 : 20). Two qPCR technical replicates were performed per sample in the iQ5 Bio-Rad thermal cycler. The qPCR program included 1 min denaturation at 95°C, followed by 60 cycles at 94°C for 5 s, 58°C for 15 s, and 72°C for 15 s. After qPCR, a melting temperature program was performed. Mean PCR efficiencies and cycle thresholds were determined from the fluorescence data using the algorithm Real-Time PCR Miner [50]. Relative gene expression of cell samples relative to SDHA was calculated using the Pfaffl method [51].

### 2.9. Reactive Oxygen Species (ROS) Quantification by Flow Cytometry

ROS (O_2_^∙−^ and ^∙^OH) generation was assayed by FCM using the fluorescent probe 2,7-dichlorodihydrofluorescein diacetate (H_2_DCF-DA), as described previously [47], which upon acetate cleavage is oxidized to fluorescent dichlorofluorescein (DCF) by hydrogen peroxide. After complexed lycopene exposure and UV-B irradiation as described before, the medium was replaced by serum-free *α*-MEM containing 10 *μ*M H_2_DCF-DA for 30 min, at 37°C in dark. Cells were washed with PBS, trypsinized with Accutase collected, and analyzed in a Coulter EPICS XL flow cytometer and ROS formation was estimated by the median fluorescence intensity (MFI) parameter using the FlowJo software.

### 2.10. Statistical Analysis

The results are reported as mean ± SD of at least three replicates/treatment. In addition, at least three independent assays were performed for each analysis. For gene expression analysis, results are reported as mean ± SE of at least three replicates from 2 independent assays. The results of all these experiments were statistically analyzed by Analysis of Variance (ANOVA with All Pairwise/Nonpairwise Multiple Comparison Procedures) using SigmaPlot 11.0 software. The differences were considered statistically significant when *P* < 0.05.

## 3. Results

### 3.1. HaCaT Cell Growth and Confluence under Normal Culture Conditions

**Figure 2:**
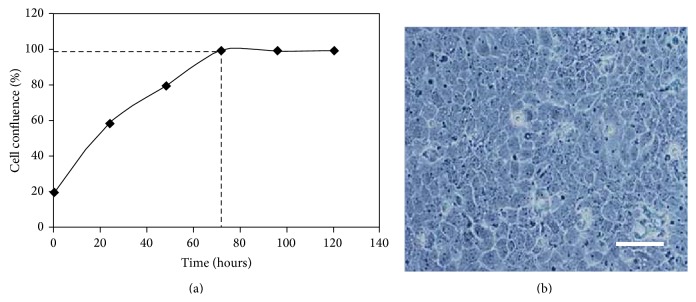
(a) HaCaT cells growth and confluence curves under normal culture conditions for 120 h. (b) HaCaT cells observed by phase-contrast microscope (100x magnification), scale bar: 20 *μ*m.

HaCaT cell growth and confluence under normal culture conditions until 120 h are represented on Figure 2(a). As it can be observed, the exponential phase extends until approximately 72 h and the full confluence can be maintained more than 1 week. The selected confluence for complexed lycopene exposure and UV irradiation in this experiment was attained at 24 h and 48 h, respectively.

Under phase-contrast microscopy, the cells displayed typical intermediate phenotype of polygonal cells interspersed with giant often multinucleated cell and single morphology (Figure 2(b)).

### 3.2. Effect of UV-B Doses on Cell Metabolic Activity by MTT Assay

**Figure 3:**
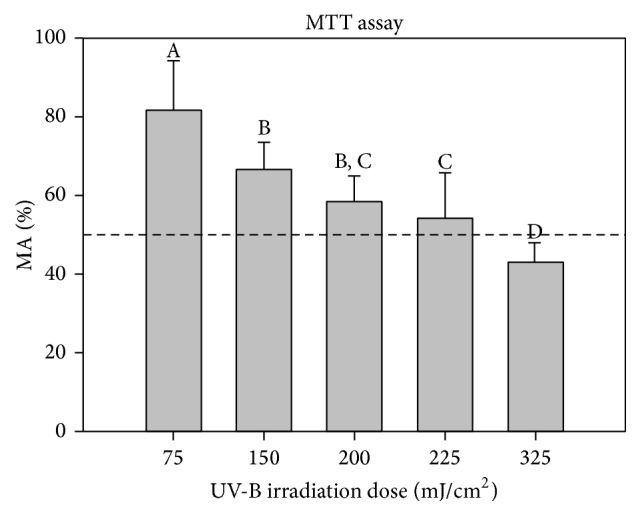
Effect of different UV-B doses on cell metabolic activity (MA) determined by MTT assay. Results are expressed as percentage (mean ± SD) of cell MA compared to nonirradiated cells. Statistical analysis: One-Way ANOVA with All Pairwise Multiple Comparisons by Holm-Sidak method: statistical differences between the samples are represented by different letters when *P* < 0.05.

As theoretically expected, increasing UV-B dose resulted in decreased cell metabolic activity (Figure 3). UV-B irradiation resulted in distinct morphological changes in HaCaT cells, as irradiated cells became round and detached from the surface of the plate.

According to MTT results, the UV-B condition tested that was near but above 50% viability was 225 mJ/cm^2^. This UV-B dose was chosen as high UV-exposure condition to analyze the maximum range of effects and response to UV exposure.

**Figure 4:**
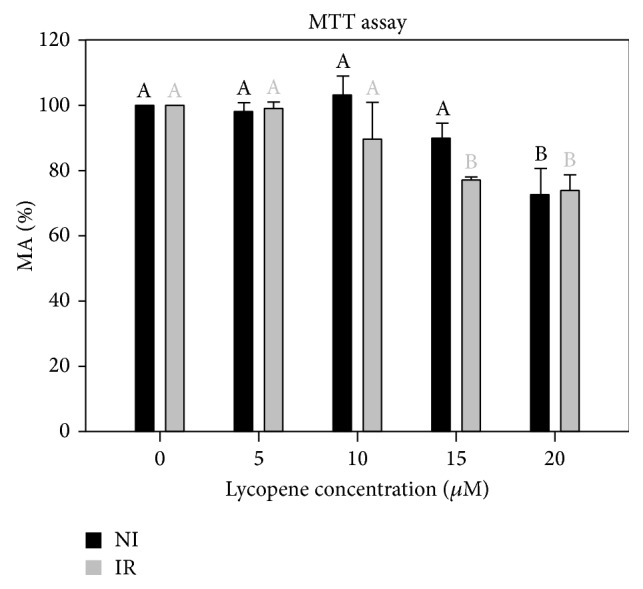
Effect of complexed lycopene preexposure on UV-B-irradiated (IR, 225 mJ/cm^2^) and nonirradiated (NI) HaCaT cells on cell MA measured by MTT assay. Results are expressed as percentage (mean ± SD of 3 independent experiments with 6 replicates each one). Statistical analysis: One-Way ANOVA with Multiple Comparisons versus Control Group (Holm-Sidak method): statistical differences between the samples within nonirradiated and irradiated groups (in respect to cells not exposed to lycopene) are represented by different letters when *P* < 0.05.

Complexed lycopene (Lyc-CD) up to 15 *μ*M did not affect the metabolic activity of nonirradiated cells, and only 20 *μ*M Lyc-CD led to significant decrease in metabolic activity in these cells (Figure 4). At doses equal to or higher than 15 *μ*M, complexed lycopene decreases the MA of irradiated cells (225 mJ/cm^2^), compared to cells not preexposed to lycopene (Figure 4).

According to preliminary MTT assays, it was also observed that concentrations of Lyc-CD higher than 20 *μ*M induced a higher decrease in MA in irradiated cells (supplementary data in Supplementary Material available online at http://dx.doi.org/10.1155/2016/8214631).

According to these results, we decided to choose an intermediate complexed lycopene concentration (10 *μ*M) whose effects have been previously established in other cell lines [52–54]. At this lycopene concentration, the cell metabolic activity was higher than 50% and was not significantly different from the nonexposed cells. Higher lycopene concentrations could decrease the cell viability, for example, due to a prooxidant effect, as suggested by a decrease in viability compared to nonexposed, nonirradiated cells.

### 3.3. Cell Cycle Analysis

**Figure 5:**
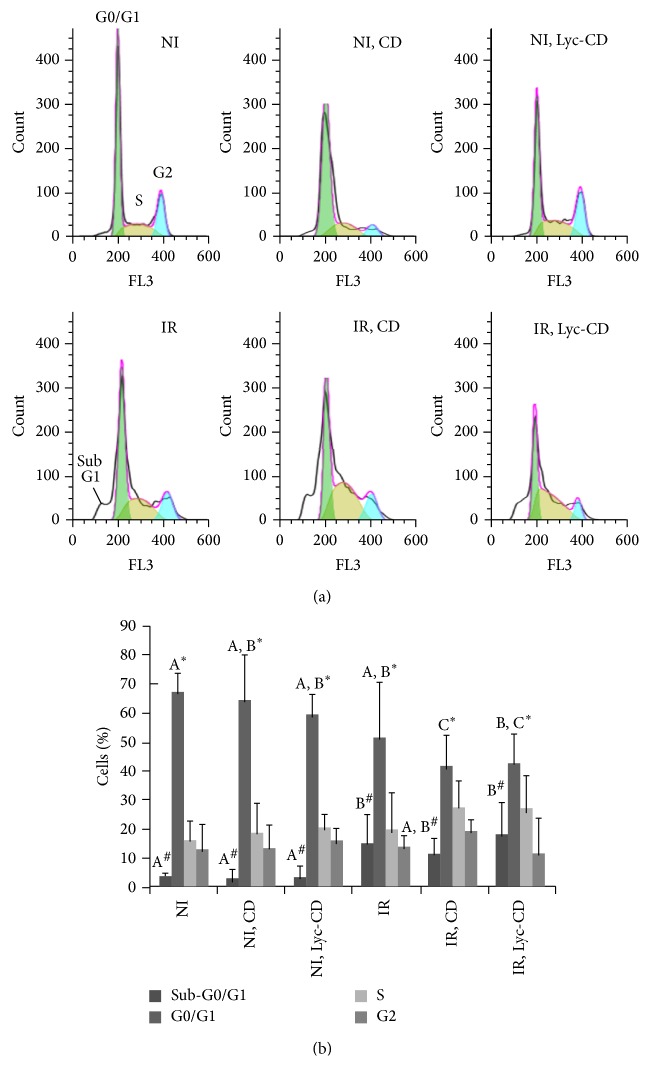
Effect of lycopene and UV-B on the cell cycle: (a) cell cycle histograms of UV-B (225 mJ/cm^2^) irradiated (IR) and nonirradiated (NI) HaCaT cells exposed to 10 *μ*M complexed lycopene (Lyc-CD) and to the respective controls; (b) cell cycle phase distribution of UV-B (225 mJ/cm^2^) irradiated (IR) and nonirradiated (NI) HaCaT cells exposed to 10 *μ*M complexed lycopene (Lyc-CD) and the respective controls, including cyclodextrin vehicle (NI, CD and IR, CD). Results are expressed as percentage (mean ± SD). Statistical analysis: One-Way ANOVA with All Pairwise Multiple Comparison Procedures: means with different letters (A, B, and C) are significantly different (*P* < 0.05). In this case, only the statistical differences between groups presenting differences (G0/G1 and sub-G1) were marked for simplification purposes. Comparison between % cells with sub-G1 amount of DNA (#) and % cells in G0/G1 phase (*∗*) is presented.

Figure 5 shows representative histograms of cell cycle of HaCaT cells after 10 *μ*M complexed lycopene exposure and UV-B irradiation (225 mJ/cm^2^). Cell cycle analysis shows that complexed lycopene exposure alone did not significantly (*P* > 0.05) affect the dynamic of cell cycle in comparison to control cells. Compared to nonirradiated and nonexposed cells, irradiation induced a decrease in the percentage of cells in the G0/G1 phase of cell cycle especially in complexed lycopene and CD exposed cells (*P* = 0.011 and 0.008, resp.) (Figure 5). Although the S and G2 phases were not significantly affected by any of the treatments, an increase in S-phase frequency can be observed.

As previously observed in Figure 5, the analysis of PI-stained nuclei by FCM cell cycle analysis showed an increase in sub-G1 subpopulations upon UVB irradiation. Exposure to CD vehicle induced a small decrease in % sub-G1 irradiated cells, compared to nonexposed, irradiated cells. Contrarily to cells exposed to CD, cell exposed to Lyc-CD did not present small decrease in % sub-G1, compared to nonexposed, irradiated cells.

### 3.4. Apoptotic Markers

**Figure 6:**
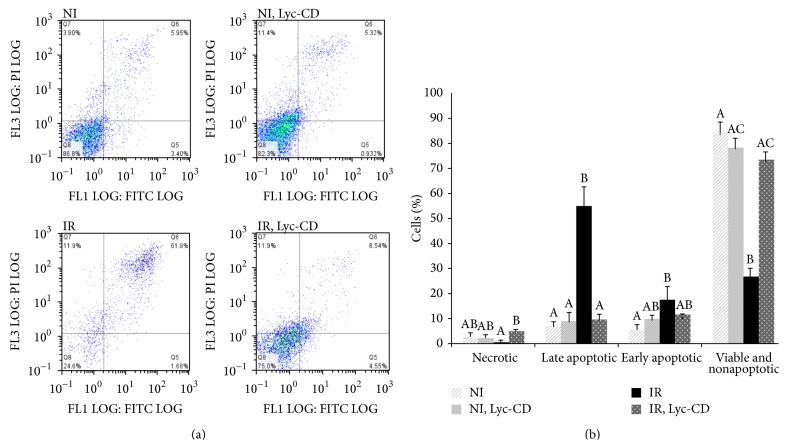
(a) Representative examples of Annexin V-FITC Dot-Plots Gating (FL1 LOG versus FITC LOG) of nonirradiated (NI) and UV-B (225 mJ/cm^2^) irradiated (IR) HaCaT cells not previously exposed or exposed to 10 *μ*M complexed lycopene (Lyc-CD); (b) results are expressed as percentage (mean ± SD, *n* = 3) of* nonapoptotic and viable cells*, Q8: Annexin-FTIC (−) and PI (−);* early apoptotic cells*, Q5: Annexin-FTIC (+) and PI (−); predominantly* late apoptotic or dead cells*, Q6: Annexin- FTIC (+) and PI (+); and predominantly* necrotic* and* dead cells*, Q7: Annexin-FTIC (−) and PI (+). Statistical analysis: One-Way ANOVA with All Pairwise Multiple Comparison Procedures (Holm-Sidak method): means with different letters are significantly different (*P* < 0.05).

Annexin V assay differentiates subpopulations that are necrotic, apoptotic, or viable. Comparative analysis of these subpopulations in irradiated (225 mJ/cm^2^) and nonirradiated HaCaT cells, treated with 10 *μ*M complexed lycopene, was performed using the Annexin V assay (Figure 6).

Within this study, a high UVB dose was used to induce cell alterations and apoptosis, but not excessive necrosis. For this study only the adherent cells were analyzed, which means that the necrotic cells in suspension were not considered in the assay results. Nevertheless, as shown in Figure 6, the main shift upon UVB irradiation was from viable to apoptotic cells, confirming that the high UVB dose used was not excessively detrimental for the HaCaT cells attached to plate and used in all the assays described here. In nonirradiated cells, complexed lycopene did not affect (*P* > 0.05) the percentage of necrotic, apoptotic, and viable cells compared to the control (nonexposed cells). Contrarily, UV-B irradiation alone increased the percentage of both early and late apoptotic cells (*P* < 0.05) and decreased the percentage of viable cells compared to the control. Contrarily to what was observed for nonirradiated cells, the Annexin V assay showed a decrease in the % cells with Annexin V positive staining (early and late apoptosis) preexposed to complexed lycopene in irradiated cells (*P* < 0.05), while the % necrotic cells increased.

Also complementary data showed that irradiated cells previously exposed to the vehicle (CD) presented, once again, results quite similar to those of nonirradiated cells (supplementary data).

### 3.5. Effect of Complexed Lycopene on the Expression of Anti- and Proapoptotic Genes

**Figure 7:**
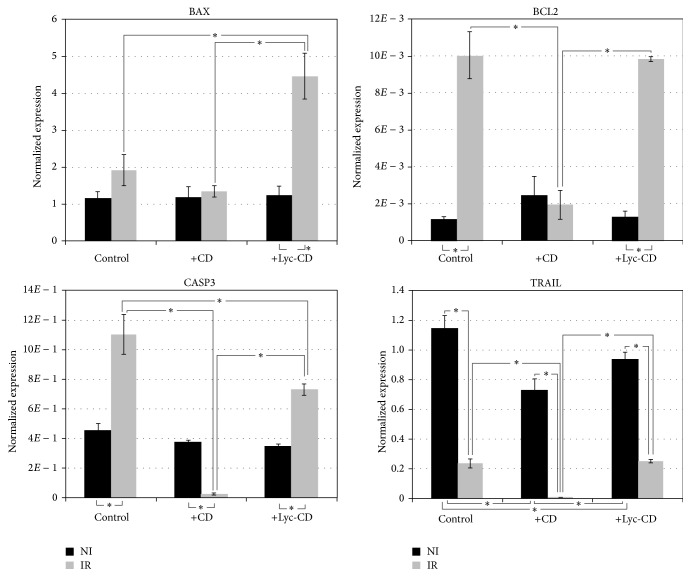
Representation of Bax, Bcl-2, caspase 3, and TRAIL gene expression values in HaCaT cells exposed to 10 *μ*M complexed lycopene (Lyc-CD) normalized to the SDHA reference gene. Cells were irradiated with 225 mJ/cm^2^ UV-B (IR) and nonirradiated (NI). Results are expressed as mean ± SEM of three technical replicates from two independent assays.

Under UVB irradiation, lycopene complex exerted proapoptotic effects compared to irradiated but not exposed cells (Figure 7). Compared to irradiated and nonexposed cells, in irradiated cells the exposure to vehicle CD inhibited antiapoptotic* BCL2* expression but did not increase proapoptotic* BAX* expression. Contrarily to this, in irradiated cells Lyc-CD increased proapoptotic* BAX* expression but did not inhibit antiapoptotic BCL2 expression. This observation points to a proapoptotic effect of Lyc-CD mediated by* BAX* upregulation. Moreover, in irradiated cells Lyc-CD showed lower caspase 3 gene (*CASP3*) gene expression compared to nonexposed, irradiated cells; however, it increased* CASP3* gene expression comparatively to CD vehicle. TRAIL is a proapoptotic cytokine secreted by many cell types; however, in this study, UVB light was found to decrease TRAIL expression.

### 3.6. Reactive Oxygen Species (ROS) Analysis

Analysis of oxidative stress induction in HaCaT cells after 10 *μ*M complexed lycopene exposure and UV-B irradiation (225 mJ/cm^2^) was performed by determining reactive oxygen species (ROS) formation by FCM.

**Figure 8:**
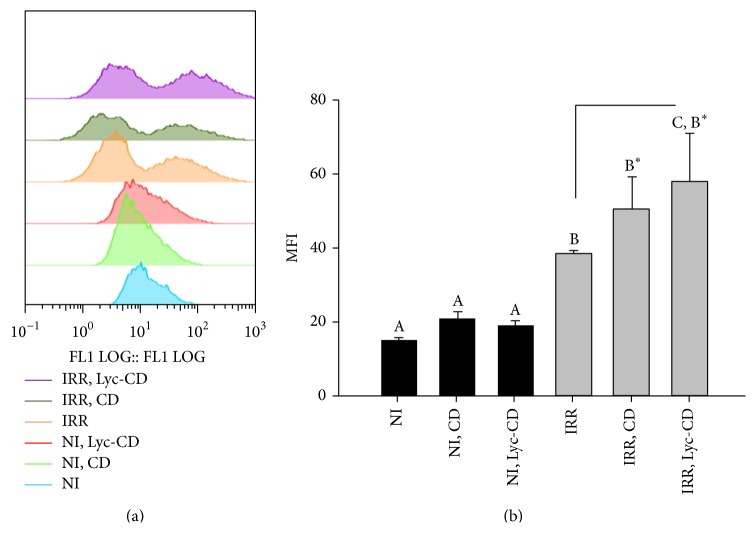
Intracellular ROS quantification: (a) FL1 histogram plots, showing the distribution of cell count versus DCF fluorescence, and (b) Mean Fluorescence Intensity (MFI) histograms results of HaCaT UV-B irradiated cells, IR (225 mJ/cm^2^), and nonirradiated cells (NI) exposed to 10 *μ*M complexed lycopene (Lyc-CD) and to the respective controls labelled with DCF-DA. Statistical analysis: One-Way ANOVA with Multiple Pairwise Comparisons: medians with different letters are significantly different (*P* < 0.05).

The results of irradiated and nonirradiated cells are represented in Figure 8. UVB irradiation induced an increase in ROS intracellular production. In fact, regarding ROS production, two cell populations were observed in irradiated cells against one in nonirradiated cells. The Median Fluorescence Intensity (MFI) from irradiated samples was statistically higher than MFI from correspondent nonirradiated cells, as theoretically expected. Complexed lycopene did not increase ROS production in nonirradiated cells. However, in irradiated cells Lyc-CD induced a significant ROS increase compared to irradiated nonexposed cells.

## 4. Discussion

In this work, we used* HaCaT cells*, a “spontaneously transformed human epithelial cell line from adult skin” which maintains full epidermal differentiation capacity. This cell line is immortal (>140 passages) but remains nontumorigenic, and it is aneuploid (hypotetraploid) with unique stable marker chromosomes indicating monoclonal origin [40]. As performed in this experimental work, further investigation needs to include studies dealing with normal cells, their transformation into malignant cells, and the association between malignant cells and the surrounding normal cells in order to determine the cytotoxicity in both cell populations.

It is noteworthy that cell culture studies are usually carried out under abnormal conditions known as “*culture shock,*” where cells are exposed to high oxygen tension and to free metal ions in the medium [27, 55]. Thus, it must always be taken into account if the study compound reacts with the cell medium. Different half-life values of lycopene under standard cell culture are reported in the literature, for example, 12–20 h [37]. Experiments were conducted with exposure time of 24 h which was sufficient to reveal the cellular effects of Lyc-CD. Lycopene chemical stability was conferred by CD complexation.

The antioxidant action of carotenoids is related to their ability to trap free radicals and quench singlet oxygen. However, depending on the redox potential of lycopene and the surrounding environment, its antioxidant activity may shift to prooxidant activity [56, 57]. In fact, the HaCaT cells medium (DMEM) contains ferric nitrate (Fe(NO_3_)_3_·9 H_2_O) and Fe(III) can react with excess neutral carotenoids, such as lycopene. Ferric ions have been proposed to degrade carotenoids by the following mechanism [58, 59]: Fe^3+^ + carotenoid ⇒ Fe^2+^ + carotenoid^*∙*+^. Although there are many Fe chelators that inhibit the reactions of Fe, oxygen, and their metabolites [60], these chelating agents may also mediate toxicity by stimulating Fe-mediated oxygen radical generation [61]. The chelating agent used for HaCaT trypsinization was ethylenediaminetetraacetic acid (EDTA) which can induce Fenton-chemistry mediated radical damage. In fact, the autoxidation of Fe(II) enhanced by EDTA was observed by others [57, 58]. Therefore, in order to prevent lycopene oxidation by Fe (III), another cell culture medium (*α*-MEM) was used without this element during cells exposure to lycopene. A normal cellular growth was observed. Furthermore, it should be also noted that the solution with the highest lycopene dose had a suitable osmolarity lower than 320 mOsm/kg (~268 mOsm/kg).

During storage,* special precautions* were taken such as the protection of lycopene from temperature, light, and air. However, during lycopene exposure, cell culture was maintained at 37°C in a humidified incubator with 5% CO_2_ atmosphere. Even considering other experimental conditions reported in the literature [62] that comprise the addition of lycopene solutions to the cell culture medium under N_2_ environment, we preferred to maintain our protocol to avoid perturbing the cell culture with other variables.

The level of lycopene normally observed in human plasma is on the order of 0.5 *μ*M even with dietary supplementation [20, 52, 53]. Therefore, for therapeutic purposes (assuming topical application) we used a range above these normal plasma levels (5 up to 20 *μ*M). According to* MTT results*, 20 *μ*M is revealed to be toxic in* in vitro* studies (Figure 4). Thus, 10 *μ*M lycopene nominal concentration was selected for further studies, supporting the selected dose used in other* in vitro* studies [52–54].

The option to use cyclodextrins for lycopene solubilization and photoprotection was based on data resulting from previous studies (Table 1) on parameters/conditions affecting the delivery of lycopene to cells, including the solubilization, stabilization, and cellular uptake by using other vehicles. In addition, similar MTT results were obtained with another vehicle (supplementary data).

HaCaT keratinocytes have a mean intermitotic time of 22–24 h* in vitro* [13] and their* cell cycle* is subjected to regulation by cyclins, cyclin-dependent kinases (CDKs), and CDK inhibitors.

UV-B irradiation activates diverse cellular responses in human cells, such as cell cycle arrest, DNA repair, and apoptosis through signal transduction pathways [15]. Previous studies have shown that UVR can cause cell cycle arrest both at G1 [63] and G2 phases [64] of cell cycle. However, in our study, irradiation did not affect significantly the percentage of cells on S and G2 phases (Figure 5). On the contrary, the percentage of cells in G1 phase decreased in irradiated cells, especially those previously exposed to complexed lycopene or the respective vehicle alone (cyclodextrins). Cyclin D regulates the transition from G0 to early G1 phase, while cyclin E regulates the transition of the cell from late G1 phase to S phase; p21 and p27 CDK inhibitors bind and inhibit the activity of cyclin E/CDK2 complex, blocking cell cycle progression in G1 phase. In fact, after UV irradiation, the half-life of the tumor suppressor (p53) appears to be extended which will induce the p21 CDK inhibitor leading to G1 phase arrest or cell death by apoptosis. G2-phase checkpoint control does not appear to be affected [65]. It has been reported that the growth inhibition of lycopene on MCF-7 breast cancer cells was also associated with decreased G1-S cell cycle progression, decreased cyclin D1 expression, and stabilization of p27 in the cyclin E-CDK complex [7, 66]. Cell cycle arrest increases the time available for DNA repair before its replication and mutation fixation processes [5]. Regarding the cell cycle results obtained with CD, it has been reported that methyl *β*-CD inhibits cell growth and induces cell cycle arrest via a prostaglandin E2 independent pathway in Raw264.7 macrophage cells [67]. Thus, a control assay with CD alone is mandatory in these studies.

*Apoptosis* is the best-characterized type of programmed cell death because of its importance in development, homeostasis, and pathogenesis of different diseases, such as cancer. Cells respond to specific apoptotic signals by initiating intracellular processes that result in typical physiological changes. Among these changes are externalization of phosphatidylserine to the cell surface, depolarization of mitochondrial membranes, cleavage and degradation of specific cellular proteins, compaction and fragmentation of nuclear chromatin, loss of cell membrane integrity, and cellular shrinkage. We studied one of the earliest apoptotic events, that is, the translocation of phosphatidylserine (PS) from the inner to the outer leaflet of the plasma membrane. Moreover, Bowen et al. [68] have found that HaCaT cell line (which harbors two mutant p53 alleles) are more susceptible to apoptosis than normal keratinocytes* in vitro*, possibly because of aberrant signaling pathways resulting from long-term culture [69, 70].

In this work, a decrease in % apoptotic cells was observed in irradiated cells preexposed to Lyc-CD (Figure 6). However, this result must be interpreted cautiously, as CD has been previously reported to deplete cholesterol from cell membranes [71, 72]. Cholesterol presence is essential for lipid raft formation and Fas-receptor activation, and this could inhibit UV-B induced apoptosis, as demonstrated by George et al. [73]. Depletion of cholesterol by methyl-*β*-cyclodextrin reduced Fas aggregation which was accompanied with a reduced apoptotic but increased nonapoptotic death of UV-B-irradiated HaCaT cells [73]. In preliminary experiments, we in fact observed that CD alone can decrease the % apoptotic cells (supplementary data). Moreover, since CD can form complexes with lipids from the plasma membrane, it might be possible that PS was shielded by CD against binding to Annexin V, a requirement for the validity of Annexin V assay. Nevertheless, cells exposed to Lyc-CD showed an increase in the Bax/Bcl2 gene expression ratio. Moreover, Lyc-CD completely reverted the inhibitory effect of CD in TRAIL expression. These results suggest a proapoptotic effect of lycopene which is not found in CD. UV-B irradiation has been shown to increase levels of* apoptosis biomarkers*, especially the proapoptotic protein Bax, thereby inducing apoptosis in skin cells. Regarding the apoptosis biomarkers, Bcl-2 family proteins can modulate mitochondrial permeability through oxidative phosphorylation during apoptosis. Changes in the Bax/Bcl-2 ratio suggest a corresponding change in mitochondrial permeability to release apoptogenic molecules from the mitochondria to the cytosol [5]. In this work, irradiated cells showed significantly higher levels of apoptosis, confirmed by independent parameters (namely, increased sub-G1 population indicative of DNA fragmentation) and binding to Annexin V, indicative of increased phosphatidylserine present in the outer leaflet of the cell membrane. From the gene expression data obtained, this means that the most relevant parameters to take into account for UVB-induced apoptosis are* BAX* and* CASP3* expression.* BCL2*, a known antiapoptotic effector was found upregulated in this study, and* TRAIL*, a proapoptotic cytokine, was found downregulated under UVB irradiation. This suggests that under the experimental conditions used, these genes have a comparatively lower predictive value for apoptosis induction by UVB. It might be a response at transcription level which does not reflect the physiological level of the irradiated cells. A more close focus on* BAX* and* CASP3* expression reveals that lycopene induced increased* BAX* expression in cells treated with the high UVB dose used in this study and this might represent an important mechanism for its therapeutic action.

Besides apoptosis, necroptosis, and necrosis or senescence, damaged keratinocytes can also be physiologically eliminated by induction of terminal differentiation. This may be readily monitored by detection of, for example, “suprabasal” keratins or cytoplasmic involucrin for late steps in epidermal differentiation; however, this type of analysis would probably be most useful for very late time points. It should be noted that more biomarkers could be also analyzed considering the huge cascade events triggered by UVR, for example, protein kinase C family which sensitizes skin to UVR [74] and others regarding the influence on intracellular calcium levels or retinol signaling which are profoundly altered upon differentiation of keratinocytes [75].

UVR results in an increased generation of* ROS* that interacts with proteins, lipids, and DNA, overwhelming the antioxidant defense mechanisms of the cells.

The epidermis is composed mainly of keratinocytes, which are rich in ROS detoxifying enzymes such as superoxide dismutase, catalase, and glutathione peroxidase and in low-molecular-mass antioxidant molecules [76]. Although skin spontaneously responds to increased ROS levels, this response may not be sufficient to prevent the progression of skin cancer [4]. Despite the extensive evidence implicating ROS in oxidative DNA damage, little is known about its involvement in DNA damage of keratinocytes, which are the most relevant cell type in nonmelanoma skin cancer. The singlet oxygen and hydroxyl radicals are the major damaging oxidative species which can be formed under normal aerobic metabolism and by certain processes including photosensitization [77]. The major DNA oxidation products include 8-oxo-7-hydro-deoxy-guanosine (8-oxodG) and 2,6-diamino-4-hydroxy-5-formamidopyrimidine [78].

Comparatively to nonirradiation, UV-B irradiation significantly altered the distribution of cells in terms of fluorescence (Figure 8). In irradiated cells two populations can be distinguished. The first peak might correspond to dead cells, which had a very weak signal of DCF (compatible with autofluorescence). In this case, the DCF might not be activated and metabolized within the cell (no fluorescence), and/or the cells might not retain the DCF because of the loss of membrane integrity. Complexed lycopene increased the ROS levels in irradiated cells. Reagan-Shaw et al. [5] have also obtained a significant decrease of SOD activity (MnSOD) after treatment with sanguinarine, enhancing UV-B-mediated oxidative stress in HaCaT cells. However, according to Onoue et al. [79], ROS data might not always provide a reliable indication for the capacity of a chemical to participant in a photogenotoxic cascade. This fact could be more realistic when irradiation is used in experiments. In fact, the photodegradation of lycopene may contribute to cytotoxicity, including oxidative damage. For example, apo-6′-lycopene and 2-methyl-hepte-6-one as well as further reaction products are formed during irradiation of lycopene [28, 80]. Nagao [81] found that oxidized metabolites of lycopene but not lycopene itself can inhibit cell growth and stimulate apoptosis in a different cell line (HL-60). In another study, lycopene in human prostate cancer cells inhibited cell growth, but the oxidized mixtures displayed markedly more potent growth inhibition [81, 82]. On the contrary, some recent studies suggested that lycopene or lycopene metabolites may, as *β*-carotene and its metabolites do, enhance carcinogenesis.

In general, similar results were obtained by Reagan-Shaw et al. [5] who suggested that sanguinarine (also a botanical antioxidant) may protect skin cells (also HaCaT cell line) from UVB-mediated damage via apoptotic elimination of damaged cells that escaped from the programmed cell death. These are clearly important observations since apoptosis is a mechanism of defense and acts by opposing the creation of damaged and preneoplastic cells and expansion to a clone. Once mutations arise, apoptosis also removes preneoplastic cells that are aberrantly proliferating due to genetic defects.

However, further investigation into the dose effect of lycopene and further understanding of the metabolism of apo-10′-lycopenoids on carcinogenesis are still needed [7]. On the other hand, studies on skin organotypic 3D-cocultures (long-lived 3D-cocultures, epiSC-markers, label-retaining cells, etc.) would be useful to have all picture considering the loss of complex interactions between the epithelium and stroma in 2D cell cultures [83]. An UV-irradiation protocol for a normal skin model or even the malignant model for both prevention and treatment approaches of skin cancer could be used [84, 85].

## 5. Conclusions

According to data obtained from all experimental assays, we demonstrate here that complexed lycopene up to 10 *μ*M does not show metabolic toxicity (MTT assay) under standard cell culture conditions. On one hand, at nontoxic dose (10 *μ*M) complexed lycopene does not affect the profile of apoptotic, necrotic, and viable cells or show cytostatic effects despite slightly increasing the ROS content. However, cells previously exposed to complexed lycopene when irradiated with metabolically damaging UV-B dose show a distinguishing switch in the dead : apoptotic : viable subpopulations compared to nonexposed irradiated cells. On the other hand, exposed irradiated cells showed a decrease in G0/G1 phase. In fact, the increased sub-G0/G1 phase and a trend for S-phase delay (even not statistically different) could contribute to this cell cycle change. In addition, an increased expression of different apoptosis biomarkers (*BAX *and caspase 3) was observed in exposed irradiated cells when compared to the respective controls. These two biomarkers were the most useful probably because those genes might be more involved in this process.

Therefore, complexed lycopene might play a corrective role or cytotoxic effect in photodamaged and preneoplastic keratinocytes, while allowing other keratinocytes to accelerate repairing mechanisms becoming viable. However, some results could be altered by the CD interference with some* in vitro* assays and also with the cells particularly after a high damage effect. Although CDs seemed to be a good candidate to vehicle lycopene in order to counterbalance the disadvantages of most solvents usually used, here we found that it was not suitable under this protocol conditions, especially UV irradiation. Nevertheless, these are interesting data regarding the CD effect on cell cultures and reveal the importance of the analysis of this control compared to cells exposed to active molecules, as it was here performed.

Future studies will continue with other exposure conditions, that is, another lycopene vehicle and a lower UV-B dose which could be set by other assays, such as TUNEL and/or detection of chromosomal breakage by Cytokinesis-Blocked Micronucleus Cytome Assay (CBMN) (besides MTT). Moreover, detection of Ki67 protein, a marker of proliferation, could be attempted. The analysis of other biomarkers and final studies using 3D skin models as previously suggested would resemble more closely the situation* in vivo*, which may give definite and clear answers.

## Supplementary Material

Supplementary Material contains parallel and preliminary studies regarding lycopene vehiculation, effect of UV-B on cell metabolic activity and apoptosis by MTT and Annexin-V assays, respectively.

## Figures and Tables

**Table 1:** Limitations of different vehicles used for lycopene cell delivery (adapted from Lin et al. [27]).

Vehicle	Limitations	References
Tetrahydrofuran (THF)	Rapid oxidation in media, leading to lycopene instability and cytotoxicity	[17, 20, 22, 28–32]

Dimethyl sulfoxide (DMSO)	Reduced solvent capacity for carotenoids (0.01 mg/mL for lycopene)	[20, 33–35]

Tween	Possible oxidation of carotenoids, after solvent drying and filtration	[31]

Micelles	Low carotenoid stabilization and increased cytotoxicity	[20, 27, 36]

Water-dispersible beadlets	Low toxicity, but also low cellular uptake, depends on chemicals that interfere in assays (e.g., hexane and chloroform)	[29, 37, 38]
