# Supplementary material for: The Effect of Lycopene Preexposure on UV-B-Irradiated Human Keratinocytes
Source: Oxid Med Cell Longev. 2015 Nov 17;2016:8214631. doi: 10.1155/2016/8214631 (PMC4664803; doi:10.1155/2016/8214631)
Supplement: Supplementary file 1 — Supplementary Material contains parallel and preliminary studies regarding lycopene vehiculation, effect of UV-B on cell metabolic activity and apoptosis by MTT and Annexin-V assays, respectively. [file 8214631.f1.pdf]

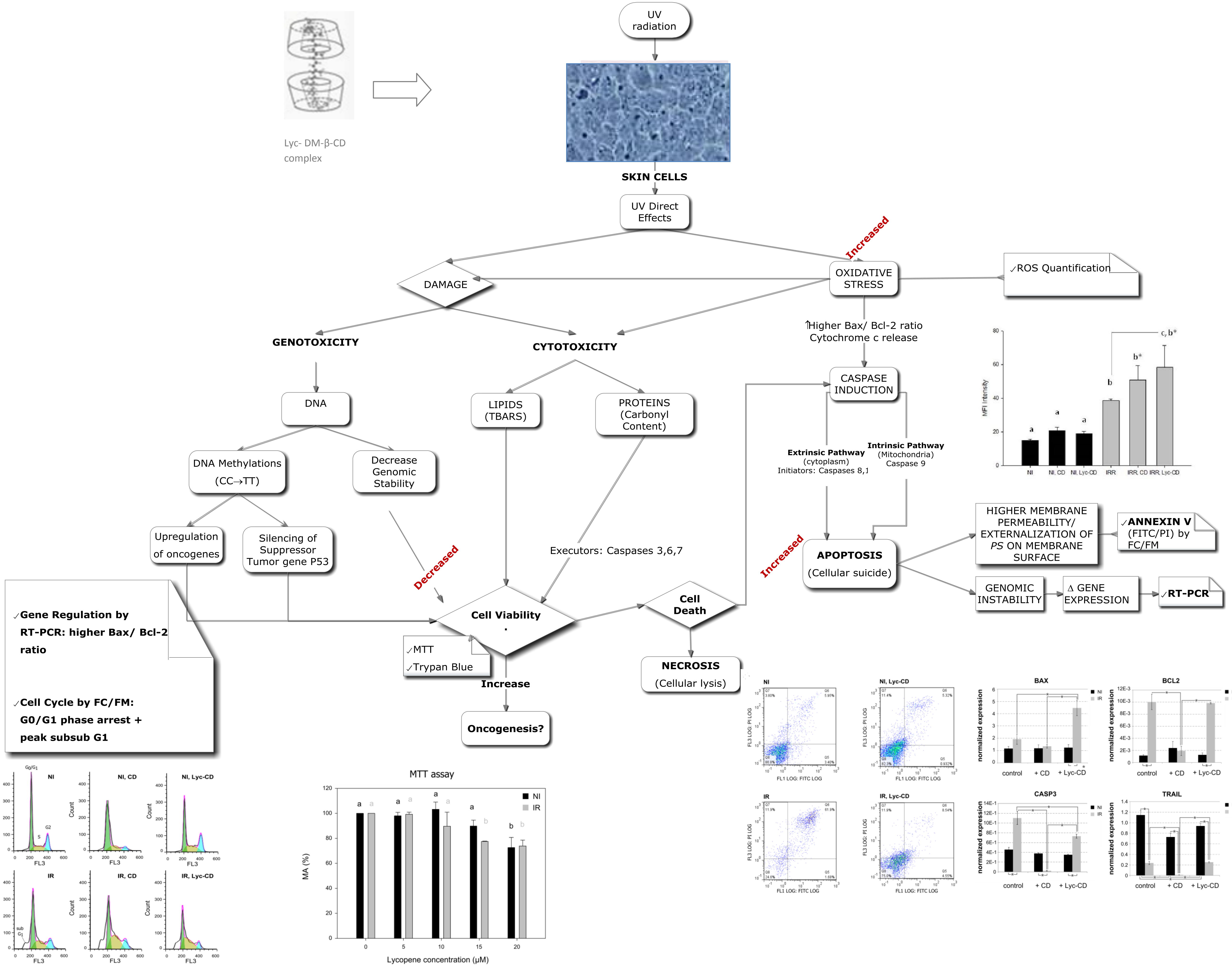

Lyc- DM-β-CD complex
UV radiation
SKIN CELLS
UV Direct Effects
Increased
OXIDATIVE STRESS
✓ROS Quantification
DAMAGE
GENOTOXICITY
CYTOTOXICITY
↑Higher Bax/ Bcl-2 ratio
Cytochrome c release
CASPASE INDUCTION
DNA
LIPIDS (TBARS)
PROTEINS (Carbonyl Content)
Extrinsic Pathway
(cytoplasm)
Initiators: Caspases 8,1
Intrinsic Pathway
(Mitochondria)
Caspase 9
DNA Methylations
(CC→TT)
Decrease Genomic Stability
HIGHER MEMBRANE PERMEABILITY/ EXTERNALIZATION OF PS ON MEMBRANE SURFACE
✓ANNEXIN V (FITC/PI) by FC/FM
Upregulation of oncogenes
Silencing of Suppressor Tumor gene P53
Decreased
Executors: Caspases 3,6,7
Increased
APOPTOSIS
(Cellular suicide)
GENOMIC INSTABILITY
Δ GENE EXPRESSION
✓RT-PCR
✓Gene Regulation by RT-PCR: higher Bax/ Bcl-2 ratio
✓Cell Cycle by FC/FM: G0/G1 phase arrest + peak subsub G1
Cell Viability
✓MTT
✓Trypan Blue
Cell Death
Increase
NECROSIS
(Cellular lysis)
Oncogenesis?
MFI intensity
NI
NI, CD
NI, Lyc-CD
IRR
IRR, CD
IRR, Lyc-CD
c, b*
b*
MTT assay
MA (%)
Lycopene concentration (μM)
IR
IR, CD
IR, Lyc-CD
FL3
Count
FL3 LOG: PI LOG
FL1 LOG: FITC LOG
BAX
BCL2
CASP3
TRAIL
normalized expression
control
+ CD
+ Lyc-CD

Supplementary Material

Vehicle for Lycopene

In another parallel work, THF/BHT diluted in FBS was also used to solubilize lycopene. Lin et al. (2007) demonstrated that FBS is likely responsible for the improved stability and cellular uptake of lycopene. In pilot experiments to screen for a suitable vehicle for lycopene, we observed that cells exposed to lycopene vehiculated in THF-BHT-FBS and irradiated also showed a decreased viability compared to non exposed irradiated cells (results not shown).

Effect of UV-B doses on Cell Metabolic Activity by MTT Assay

According to preliminary MTT assays, it was observed that concentrations of Lyc-CD higher than 20 µM induced a higher decrease in MA in irradiated cells. In these preliminary assays, exposure to the vehicle (CD) alone did not significantly alter the MA of non-irradiated cells.

Apoptotic markers

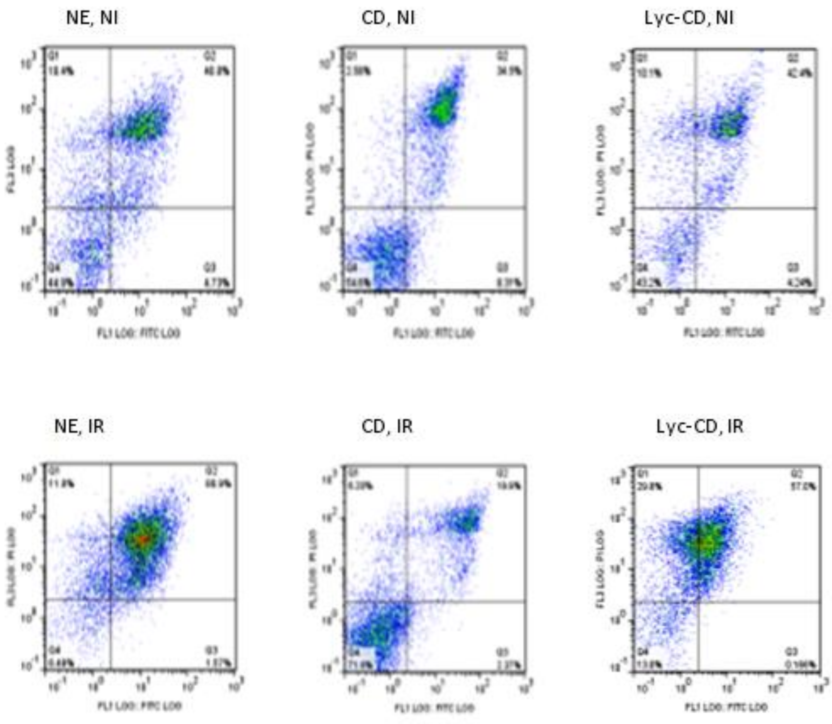

Representation of Annexin V-FITC Dot-Plots Gating (FL1 LOG *vs* FITC LOG) of UV-B (225 mJ/cm²) irradiated (IR) and non irradiated (NI) HaCaT cells previously exposed to 10 µM complexed lycopene (Lyc-CD) and only to the vehicle (CD) or non exposed (NE) correspondent to a complementary experiment.
